# Developing Indicators of Age-Friendliness in Taiwanese Communities through a Modified Delphi Method

**DOI:** 10.3390/ijerph192114430

**Published:** 2022-11-04

**Authors:** Jo-Ying Huang, Hui-Chuan Hsu, Yu-Ling Hsiao, Feng-Yin Chen, Shu-Ying Lo, Tzu-Yun Chou, Megan F. Liu

**Affiliations:** 1 School of Gerontology and Long-Term Care, College of Nursing, Taipei Medical University, Taipei 11031, Taiwan; 2 School of Public Health, College of Public Health, Taipei Medical University, Taipei 11031, Taiwan; 3 Research Center of Health Equity, College of Public Health, Taipei Medical University, Taipei 11031, Taiwan; 4 Department of Nursing, Center of Geriatric Care Resource, College of Medicine, Fu Jen Catholic University, Taipei 242062, Taiwan; 5 Division of Community Health, Health Promotion Administration, Ministry of Health and Welfare, Taipei 103205, Taiwan

**Keywords:** age-friendly, age-friendly community, indicator, modified Delphi method

## Abstract

This study developed indicators of age-friendliness for communities in Taiwan that conform to international standards by referring to the World Health Organization Checklist of Essential Features of Age-Friendly Cities and Taiwan’s existing indicators. The first stage of the research was based on the WHO’s framework and involved a literature review to identify candidate indicators. In the second stage, experts’ opinions were collected through a modified Delphi method, and the indicators were screened and revised on the basis of their importance, community enforceability, and generality. The third stage focused on practical feasibility. External parties were invited to offer their opinions regarding the indicators, which were adjusted accordingly. After three rounds of review and re-examination based on the modified Delphi method, the final set of indicators comprised five core indicators and five optional indicators. These indicators can be used to monitor various aspects of communities and determine their age-friendliness.

## 1. Introduction

The World Health Organization (WHO) identified active aging as a core value in 2002 and indicated that all countries should address population aging through policy [1]. The WHO then published “Global Age-Friendly Cities: A Guide” in 2007, which identified eight crucial factors for cities to create appropriate infrastructure for older adults [2]. The WHO also released “Measuring the Age-Friendliness of Cities: A Guide to Using Core Indicators” in 2015, which revealed essential factors for older adults to manage physical, psychological, and social change. Health care, the built environment, social services, cultural attitudes, equity, and inclusion affect social participation among older adults. The WHO’s guide provides a framework and a set of core and supplementary indicators of a city’s age-friendliness and the effectiveness of related interventions. The core indicators enable comparison across time and space, whereas the supplementary indicators can be used situationally. Past studies have found that age-friendly cities may consider older people’s frailty, self-rated health, and well-being [3,4,5]. These indicators can be used to encourage community engagement, empowerment, advocacy, and collaboration and to create inclusive and accessible communities for older adults, thereby promoting their health and well-being [6].

To measure the achievement and changes in age friendliness, researchers have tried to develop age-friendly city indicators. Most of the studies used individual-based indicators [7,8,9,10,11,12,13,14,15]. Cao et al. [8] suggested indicators in two domains: the built environment and the social environment. Choi [9] suggested 66 indicators under the eight domains of the WHO’s age-friendly city. Chiu et al. [10] categorized the key themes of age friendliness from the perspective of older adults: transportation, public spaces, welfare services, housing, and workplace discrimination. Some studies developed age-friendly city indicators that correspond to the eight domains of the WHO’s age-friendly city framework [4,12,13,14,15]. In recent studies, the inclusion of technology and information and communication technology (ICT) is also considered as a part of age-friendly city [16]. The indicators were all individual perceived measures, such as “there are enough opportunities to meet people in my neighborhoods”, and “when I am ill, I receive enough care that I need”. However, these indicators are usually numerous and based on an individual’s perception. The real situations in the community were not reflected in the indicators.

Only a few studies have used community-unit indicators to measure age friendliness [4,17,18,19]. Kano et al. [17] developed community-unit based age-friendly city indicators and piloted in 15 communities from 12 countries. The data were available in the existing data or surveys, and the community-based indicators covered the domains of the physical environment, social environment, impact, and equity domains. The example indicators were “proportion of older people living in a household with a disposable income above the risk-of-poverty threshold” and “Proportion of streets in the neighborhood that have pedestrian paths which meet locally accepted standards”. These indicators may reflect the domains of age friendliness, and the data were already available. However, there are some limitations to the application of these indicators. For example, the data were from communities; thus, the existing survey may be too old to reflect the status quo, or the communities may not have the ability to collect or to calculate the number (such as the equity indicators). Davern et al. [18] also developed 33 spatial indicators to respond to the eight domains of the age-friendly city from the WHO’s framework. These spatial indicators emphasized the physical environment, such as walkability for transportation, access to public transport stop within 400 meters, proportion of household in the bottom, and access to neighborhood community centers. Although these indicators are straightforward and reliable, they only cover spatial indicators in the physical environment. Wong et al. [4] applied both individual-level subjective indicators as well as objective neighborhood indicators; the objective neighborhood indicators were obtained from the Geographic Information System to the 1-kilometer environment around the individuals, such as open space, road density, and governmental services. In addition, qualitative methods, such as photoproduction, may also be applied to examine the age-friendliness of a city [19], which can be useful in examining the built environment and devices of age-friendly services in the community.

Although the age-friendly city indicators have been developed in previous research, there are some weaknesses in applying these indicators directly by the government or the communities. First, it is common that different areas use different indicators [7]. The strength is that unique characteristics for the areas can be considered, but this prevents comparison across areas or implementation of a universal policy for the central government. Second, most of the studies suggest many indicators, which may be useful in academic research, but may not be practical in health policy or conduction by local governments. Based on the eleven cases in the Global Network of Age-friendly Cities and Communities, involving key actors in age-friendly city programs or work is necessary [20] It would be better if the key actors are involved in the construction of community-based age-friendly indicators. Third, data collection by the local government or districts may be hard for untrained personnel. The WHO has suggested principles to develop indicators for age-friendly cities: measurable, valid, replicable, sensitive to change, disaggregation possible, aligning with local goals and targets, linked to action, within local influence, easy to collect, and socially acceptable [21]. If the purpose of the suggested indicators is for policy to link to actions and within local influences, the indicators should be simple and easy to collect, and the data reliable. An easy-to-implement tool with fewer indicators is more likely to be applicable in communities or districts.

### Background in Taiwan

Since 2010, the Health Promotion Administration of the Ministry of Health and Welfare in Taiwan has advanced initiatives to create age-friendly cities and integrate into the international community. It launched the Age-Friendly Environment Monitoring Program in 2015 [21] and developed indicators of the age-friendliness of Taiwan’s cities that match international standards and can be applied nationwide. The indicators were finalized in 2020 after several rounds of discussion and revision by an expert panel in accordance with the WHO’s framework. The indicators were based on those already in use in cities across Taiwan and on others developed in Canada, Japan, Hawaii, and Europe; they also comprised both core and supplementary indicators [22].

City- and individual-level data were used to develop the indicators, and the threshold for the data sources was high. In Taiwan, the availability of resources differs by community, which has created a gap in software and hardware between urban and rural communities. As a result, developing indicators of age-friendliness may be difficult because of the challenges in obtaining data and satisfying certain criteria; this prevents the evaluation of age-friendliness interventions. Indicators should thus be developed using the community as the smallest unit. Such indicators can help communities track each stage of an intervention, evaluate its effectiveness, and determine whether it accomplishes the desired goals. In this study, the community is defined as the administration unit under cities, i.e., the districts and neighborhoods (Li, or ‘里’ in Chinese). On one hand, the age-friendly city policy is promoted by the Health Promotion Administration of the Ministry of Health and Welfare in Taiwan, and the basic unit of primary care and public health at the local level is at the district level. On the other hand, the district is also the basic administration unit for all policy planning and administration. For other community services, the neighborhood (Li) is the smallest unit for administration, and they are more accessible to the residents in the neighborhoods. The purpose of this study was to develop community-level age friendliness indicators, and thus to explore strategies enabling communities to use quantitative systems to adjust interventions and utilize their resources appropriately.

## 2. Materials and Methods

**Figure 1:**
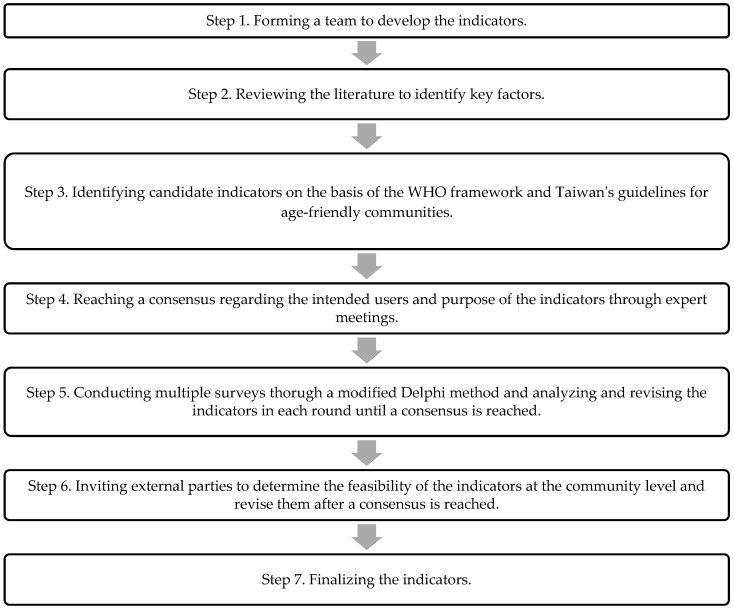
Process of developing the indicators.

This study conducted a preliminary literature review, held a meeting among experts, and synthesized their opinions in three stages (Figure 1).

### 2.1. Step I: Forming the Team

The team comprised local government officials involved in matters related to age-friendliness, scholars of policy issues related to age-friendly cities and communities, physicians experienced in community medicine and geriatric medicine, and professors specializing in indicators and data analysis. We reviewed 20 potential participants, and the final team comprised 4 scholars and 3 elder-care physicians.

### 2.2. Step II: Systematic Literature Review

A systematic literature review of age-friendly city indicator research or application cases in cities worldwide was conducted first [2,8,10,11,12,13,14,15,16,21,23]. The existing literature and government documents in Taiwan about existing age-friendly city indicators were also reviewed [6,22,24,25]. Greenfield et al. [26] introduced 3 theories to explain community collaboration in age-friendliness: asset-based community development, strategic action, and collective impact. All these theories emphasize mobilizing and collaborating with communities. Thus, the age-friendly city indicators should be acceptable and practical for the community stakeholders. Their opinions should be considered. In fact, in the experience of Taiwan’s age-friendly city programs, stakeholders’ inputs are essential, and the integration top-down and bottom-up perspectives is necessary in the development of community-level indicators [27]. In addition, although Taiwan is a small island, the heterogeneity across cities/countries and districts are various. Some cities cover offshore islands and remote areas in the mountains, and lifestyles of aboriginal ethnicity groups need to be considered [28]. The suggestions in the community-level indicators should be flexible to allow the variance across communities.

Taiwan’s indicators were mainly based on the WHO’s global city age-friendliness indicator framework and focused on core indicators. To ensure that indicators are effective, a hierarchical database must be created [22,24]. Most policies and systems to increase age-friendliness had only been implemented at the city level and through a top-down approach. This created difficulties for local health bureau personnel and elderly people in the community. Because cities and communities differ in terms of resource availability and the types of challenges they encounter, one set of standards for age-friendliness cannot be applied to both. In addition, a comparison of basic infrastructure, living service facilities, and medical resources in various regions revealed a large developmental gap between urban and rural areas; rural areas, especially disadvantaged towns and remote areas, had few living service facilities. This gap also applies to transportation, outdoor space, and buildings [25].

### 2.3. Step III: Identifying Candidate Indicators

After the systematic literature review, this study referenced international indicators of age-friendliness in communities to strengthen Taiwan’s guidelines for age-friendly communities [29]. We also modified the evaluation form suggested by the Public Health Agency of Canada [23] to design the contents for the indicator descriptions.

We then determined the objectives and operational definitions of the indicators and evaluated the feasibility of data collection for frontline personnel at the community level. Items amenable to monitoring were then identified as candidate indicators (Supplementary Table S1).

### 2.4. Step IV: Reaching a Conensus

#### 2.4.1. Expert Meeting

To avoid the time-consuming process of distributing multiple questionnaires, which may have indirectly affected the results, we explained our objectives and the purpose of the indicators to the team during the expert meeting [30] and conducted three expert opinion surveys. The team reviewed each candidate during the meeting to ensure that they would be useful and effective at the community level and that they would enable the bottom-up development of age-friendly communities. The team discussed the role of users, indicator standings, and the indicators’ purpose. During the meeting, the team concluded that the indicators should be explained in plain language to facilitate usage at the community level and conform to international standards. For this reason, all eight factors from the WHO framework were retained. The indicators were then developed through a modified Delphi method.

#### 2.4.2. Modified Delphi Method

The modified Delphi method was used to analyze the experts’ opinions, prevent disagreement, and ensure consensus was reached [31]. In the first survey, importance, practicability for the community (observability and ease of data collection), and applicability (to urban and rural areas) were the scoring criteria. In the second survey, potential limitations of intended users of the indicators were considered, and the team concluded that the number of indicators should be small and that the participants should be allowed to adjust. The indicators were then categorized as either core or optional. In the third survey, the scoring criteria were used to determine whether the purpose and definitions of the indicators were appropriate. After each round, items were added, deleted, or adjusted on the basis of the experts’ opinions. The team reviewed, discussed, and revised the indicators through three rounds of questionnaire surveys based on a modified Delphi method. Specific terms and concrete examples were added during the revision process. Ambiguous indicators were redefined, and inappropriate indicators were removed. These differences were noted, and the scores and experts’ comments were presented with the names of the experts kept confidential to facilitate scoring and review in the next round. A quantitative analysis of the third survey revealed that the team’s opinions had reached consistency, and subsequent surveys were canceled with the consent of the team. The three rounds lasted approximately 3 months. All team members remained throughout the process, and the questionnaire response rate was 100% for each round.

### 2.5. Step V: Meeting with External Committee Members and the Local Health Department

On one hand, the community-level indicators are expected to be applied by the central government (Health and Promotion Administration) and local governments, so vertical integration and directing the community’s attention to problems related to age-friendliness through administrative initiatives are crucial to creating age-friendly communities. Thus, the results must be also reviewed and consensus obtained from the government. On the other hand, the actors are the community stakeholders. The indicators must consider practicability in collecting the data and sensitivity for actions in the community. For this reason, the operational definitions and data sources were revised to suit the context of the community and prevent challenges. Thus, the external committee members included the central government (Health Promotion Administration) officials, local government staff, community representatives and not-for-profit organization representatives.

After the team identified the preliminary indicators, a meeting was held with 17 local health personnel from four counties and cities and four additional experts. These participants provided opinions as frontline staff at the community level, discussed problems that might arise when the indicators are used, and offered suggestions. The indicators were adjusted and finalized on the basis of the consensus reached during the meeting.

## 3. Results

### 3.1. Consensus Regarding Expert Questionnaire Based on the Modified Delphi Method

This study performed both quantitative and qualitative analyses on the team’s opinions regarding each indicator and suggestions for revision. In the quantitative analysis component, for each round of the Delphi process, indicators were scored on a scale of 1 to 5 points, representing “very inappropriate” to “very appropriate,” respectively, and quantitative statistical analysis was performed on the resulting scores. The appropriateness assessment was based on means and modes. A mean larger than or equal to 4 or a mode of 5 indicated that the team considered the indicator to be important, that the indicator was observable in the community, that the data were collectible, and that the indicator was applicable to both urban and rural areas. The consistency evaluation was based on standard deviations. A standard deviation less than or equal to 0.50 indicated high consistency, whereas a standard deviation between 0.50 and 1.00 represented moderate consistency. When an indicator had low consistency, the team decided whether to revise or delete it. In the qualitative analysis component, before each survey, the modifications during the previous round were explained to the experts. The questionnaires had a comment column for each indicator where experts could note questions or opinions. After each questionnaire, the investigator adjusted or retained the indicators and responded to the experts’ comments.

#### 3.1.1. First Round

In the first round, the mean value for importance was between 3.50 and 4.83. Of the 42 indicators, 6 were highly consistent, 25 were moderately consistent, and 11 had low consistency. The mean community practicability score ranged from 2.50 to 4.67. Only 1 of the 42 indicators was highly consistent, 23 were moderately consistent, and 18 had low consistency. The mean applicability score was between 3.00 and 4.67. Only 1 of the 42 indicators was highly consistent, 22 were moderately consistent, and 19 had low consistency. The team concluded that although some indicators were useful, they would be difficult to apply in remote communities, and the operational definitions were not specific, which might negatively affect the data because of differences in interpretation. The team suggested that the terminology be made more concrete and supplemented with examples to enable users to understand the indicators and how to use them.

#### 3.1.2. Second Round

In the first round, the indicators were required to score 4.00 for at least two of the selection criteria, namely importance, community practicability, and applicability; otherwise, they would be deleted. After the first round, the number of indicators was 22. The indicators were revised on the basis of the results of the first round to create the second questionnaire. The mean scores for the second round ranged from 3.00 to 4.67, and 17 of the 22 indicators had moderate consistency, whereas 5 had low consistency. The team continued to revise the indicators’ definitions (data sources, numerators, and denominators) to make them more specific. Inappropriate or repetitive indicators were eliminated. Consistency increased between the first and second rounds.

#### 3.1.3. Third Round

In the third round, indicators with a mean score of 4.50 or more and a mode of 5 were considered core indicators. Those with a mean score between 4.00 and 4.50 were optional indicators, and those with a mean score lower than 4.00 were deleted. The number of indicators retained from round two was 10: 5 core indicators and 5 optional indicators. Indicators were adjusted and deleted on the basis of the second round to produce the third questionnaire. The mean importance score was between 4.83 and 5.00, and all 10 indicators were highly consistent, with a mode of 5. The mean community practicability scores ranged from 4.50 to 4.83; seven indicators were highly consistent, three were moderately consistent, and no indicators had low consistency. The mode was 5 for both groups. Applicability scores ranged from 4.67 to 4.83. Eight of the indicators were highly consistent, two were moderately consistent, and the mode was 5 for both groups.

A comparison of the results of rounds two and three revealed that the appropriateness and consistency among the team’s opinions increased considerably, eliminating the need for subsequent rounds (Table 1).

### 3.2. Outcomes of External Committee Meeting

During the meeting, the external committee and local health officials agreed on the practicability and importance of the indicators, whereas perspectives regarding administrative resources and the authorities responsible for observing targets varied. Because community personnel can encounter obstacles during data collection, the committee suggested that the indicators allow for adjustment. In addition, the committee suggested that the operational definitions be clear to ensure consistent interpretation among community personnel in each region and thus consistent outcomes. The face validity and the content validity were confirmed by the external committee meetings. On the basis of the conclusions of the meeting, the names of two indicator domains and one indicator, the descriptions of one indicator, six numerators, and four denominators, the meaning of three indicators, and the implications of two indicators were revised.

### 3.3. Results

The indicators were revised three times through the modified Delphi method. The process began with 42 candidate indicators, which decreased to 22 after the first round, 10 after the second round, and ended with 10 after the third round. The indicators were further examined at the external committee meeting, where some indicators were revised. The final 10 indicators (Table 2) were finalized by incorporating the opinions of the experts and frontline personnel at the community level (Supplementary Table S2). 

The core indicators are essential to creating age-friendly communities. Frontline personnel at the community level can collect data and use the indicators regularly to evaluate and monitor various locations. The optional indicators are based on respect for the diversity of local communities. Communities use certain optional indicators to collect relevant data. Because the level of urbanization, resources, and city development are different across cities/districts, uniform indicators for policy monitoring and control would be hard to implement and unacceptable. Thus, we suggested allowing for flexibility in these optional indicators for different areas. These optional indicators enable frontline personnel at the community level to quantitatively determine the benefits of age-friendly interventions by analyzing data and to effectively utilize administrative and medical resources in the community to maximize benefits.

The five core indicators and the corresponding domains of age-friendly cities were the proportion of public restrooms that are clean and accessible (outdoor spaces and buildings); availability of public transportation information (transportation); satisfaction with bus drivers’ attitudes (transportation); proportion of age-friendly venues (social participation); and satisfaction with age-friendly counter services at government agencies (communication and information). Three of them were related to physical environments, and two indicators (driver’s attitudes and government service satisfaction) were related to social environment.

The five optional indicators and the corresponding domains of age-friendly cities were: the proportion of public buildings that have accessible entrances and exits (outdoor spaces and buildings); timely removal of road of obstacles (transportation); proportion of care services provided at community care centers (community support and health services); visual friendliness satisfaction with government agencies (communication and information); and amount of information disseminated to older adults through channels (communication and information).

The data sources of these 10 indicators may come from existing facility statistics, government documents, and population-based surveys. The operational definition, data sources, meanings, and implications are described in Table 2. To assure the feasibility of application of these indicators, the authors also developed a tool booklet along with the indicators to describe how to conduct a survey or obtain these data if the officials or personnel in the districts were not familiar with these research methods.

## 4. Discussion

This study developed age-friendliness indicators at the community level using a modified Delphi method for the case of Taiwan. Ten age-friendliness indicators for communities are suggested, including five core indicators and five optional indicators. Most age-friendly policies and systems only apply to the county and city levels, resulting in a low awareness of such programs among older adults in the community. At the same time, most studies measure age-friendliness at the individual level, and thus community-based collaboration and actions are not directly linked. This study focused on the community level as the starting point to develop an age-friendly community and lay the foundations for a support system. 

### 4.1. Domains and Indicators of Age Friendliness

The domains of the community-level age friendliness indicators developed by this study respond to the WHO’s framework in outdoor spaces and buildings, transportation, social participation, community support and health services, and community and information. Taiwan’s city-level age-friendliness indicators correspond to six domains: outdoor spaces and buildings, transportation, social respect and social inclusion, employment and civic participation, community support and health services, and community and information [22]. Thus, our community-level indicators overlap with the four domains of age-friendly city indicators at the city level. At the community level, the focus of age-friendliness would be more related to community infrastructure and community services. The domain of employment and civic participation is more related to policy at the city level, and respect and social inclusion is more related to the culture at the national level. The community is defined as a district or neighborhoods in this study. The district under a city is the basic administration unit for all policy planning and administration. Each district has a public health center to provide public health services and primary care for the people in this district. To promote a cross-department age-friendly policy or practices, the district is the most suitable agency in collaborating the partnership with private sectors or community actors. The district has the authority to address respect and social inclusion in government departments, and to promote social inclusion by encouraging community support and activities. At the neighborhood (Li) level, they usually can plan and conduct a small number of community services, and can provide immediate support for disadvantaged older adults. Thus, developing community-level indicators may help community stakeholders and members to focus on what they can do to promote age-friendliness.

Among the 10 suggested indicators, five indicators belong to physical environments (proportion of public restrooms, proportion of age-friendly venues, accessible proportion of public buildings, timely removal of road of obstacles, visual friendliness satisfaction with government agencies), and five indicators belong to social environments (availability of public transportation information, satisfaction with bus drivers’ attitudes, satisfaction with age-friendly counter services at government agencies, proportion of care services provided at community care centers, and information disseminated to older adults through channels). Previous studies usually more focus on physical environmental indicators [4,18]. Spatial or built environmental indicators are often objective and easy to obtain from existing data. However, the social environmental indicators emphasize the software and functioning of age friendly policy and programs. The social environment indicators of age friendliness are highly suggested for inclusion.

In addition, the age-friendly indicators in this study focus more on health domains than in other dimensions. It is because the central government body responsible for implementing age-friendly cities is the Health Promotion Administration, Ministry of Health and Welfare. It is expected that the departments of health in local governments will take the initiative in age-friendly city policies and collaborate with different departments in local governments and with community members. The staff in the health departments have usually been well-trained and are familiar with working with communities. However, dimensions other than health may not be taken as the priority in this development.

### 4.2. Indicators at Different Levels 

In the literature, most of the age-friendly city indicators are either designed for the city level (for the local government policy) or at the individual level. Only a few research designed community-based indicators to measure age-friendliness [4,20].

The strengths of the individual-level indicators include the possibility of collecting data directly from older citizens about their perception and experiences, and a survey at the individual level may provide information when existing area-level data are unavailable. When the age friendliness indicators are applied to show the effects of the actions of communities, community-level indicators are more useful to monitor the status quo and any changes. In our study, we used a community as the unit to design the indicators, and provided implications of the actions for this indicator. In addition, the community-level indicators are required for the central government (Health Promotion Administration) to monitor the effectiveness of age-friendly city policy. To assure feasibility and to prevent resistance from the communities, health departments of local governments and community stakeholders were also invited to provide comments and suggestions to form these indicators. The central government in Taiwan tends to integrate both top-down and bottom-up perspectives in the age-friendly city policy [24,27].

### 4.3. Limitations

There are some limitations in this study. First, the original project planned to pre-test the indicators in some communities and collect qualitative opinions for refining the indicators, and then to provide an educational program for local government staff and communities about the meaning and application of these indicators. However, the COVID-19 pandemic influenced the routines of local governments and communities. The outbreaks inhibited the original program, the delivery had to change to online pre-recorded videos as the educational materials, and the pre-test could not be conducted after the development. Second, the project planned to implement the indicators in all the communities in Taiwan when the indicators were developed and confirmed. Due to COVID-19, we were unable to include older adults’ opinions in the indicator development process; also, there was no chance to implement the data collection in the communities at that time. The criterion validity and construct validity were unconfirmable without the empirical data. We expect the empirical data will be collected in the nearest future. Third, the indicators were constructed based on the existing resources and age-friendly policies in Taiwan, and thus the indicators may not be generalized to apply on other places. However, age-friendly city indicators should meet the resources, culture, real-life situations, and policies for each city or country, especially at the community level to promote following actions.

## 5. Conclusions and Recommendations

### 5.1. Conclusions

This study used a modified Delphi method to apply the professional knowledge and experience of experts through group discussion. The experts exchanged views and discussed the indicators and integrating elements of dementia-friendliness and compassionate care during the meetings. Multiple questionnaires were conducted to review and revise the indicators and eliminate inappropriate indicators. External parties and local health personnel were invited to examine the feasibility of the indicators and potential problems for users; we incorporated their opinions into the final set of indicators. These age-friendly indicators can be measured quantitatively, and can be used over a period of time to evaluate the status of age-friendly implementation in each community later.

### 5.2. Recommendations

#### 5.2.1. Improving the Indicators

This study only developed preliminary indicators. Including too many indicators prior to the construction of a database was avoided to prevent aversion toward the indicators in the community. The indicators cannot be fully developed through one study, and their validity must be examined over an extended period. The indicators can be improved by adding a social participation dimension and local elements.

#### 5.2.2. Creating a Database

The systematic literature review and the process of developing the indicators indicate that to be effective, indicators must be based on reliable data sources. However, the intended users of the indicators are personnel in county and city health bureaus and frontline personnel at the community level; obtaining the required data for the indicators may be difficult. Local governments should create databases to increase accessibility to data at the community level.

#### 5.2.3. Improving Frontline Personnel’s Skills

Many community-level personnel are committed to improving age-friendly communities by collaborating with central authorities and the local government. However, this relies on the leadership of the health department. Community-level personnel may require more expertise and experience with systematic work to improve their communities. To improve their skills, teaching materials based on the indicators should be developed.

#### 5.2.4. Promoting the Indicators

These newly developed indicators should be promoted to raise awareness about age-friendly communities. Age-friendly policies and welfare systems have mostly been promoted through rigid systems, preventing them from reaching communities. For this reason, story-based marketing techniques should be used to promote the newly developed indicators.

## Figures and Tables

**Table 1:** Experts’ evaluations of indicators of age-friendliness of Taiwan’s cities based on a modified Delphi method.

Domain/Indicator Number	First Round									Second Round			Third Round								
	Importance			Community Enforceability			Generality			Development Possibilities			Importance			Community Enforceability			Generality		
	SD	Mo	Mean	SD	Mo	Mean	SD	Mo	Mean	SD	Mo	Mean	SD	Mo	Mean	SD	Mo	Mean	SD	Mo	Mean
Outdoor Space and Buildings																					
1.1	0.52	4	4.33	0.63	4	4.00	0.63	4	4.00	0.63	3.00	3.00									
1.2.1	0.55	5	4.50	1.21	5	3.67	1.03	3	3.33												
1.2.2	0.55	5	4.50	1.38	5	3.50	1.21	4	3.33												
1.3	0.52	4	4.33	1.05	4	3.50	0.98	3	3.17												
1.4	0.55	5	4.50	0.75	4	4.17	1.10	4	4.00	1.03	5.00	4.33	0.41	5.00	4.83	0.84	5.00	4.50	0.52	5.00	4.67
1.5	0.55	5	4.50	0.52	4	4.33	0.52	4	4.33	0.82	5.00	4.67	0.00	5.00	5.00	0.82	5.00	4.67	0.41	5.00	4.83
Transportation																					
2.1	0.41	4	4.17	0.63	4	4.00	0.63	4	4.00	0.63	3.00	3.00									
2.2	0.41	5	4.83	1.63	5	3.33	1.21	3	3.67												
2.3	0.84	5	4.50	0.84	5	4.50	0.84	5	4.50	0.82	5.00	4.67	0.41	5.00	4.83	0.82	5.00	4.67	0.52	5.00	4.67
2.4	0.82	5	4.33	0.98	5	4.17	1.26	5	4.00	0.63	3.00	3.00									
2.5	0.52	5	4.67	0.75	4	4.17	1.10	4	4.00	1.03	5.00	4.33	0.41	5.00	4.83	0.41	5.00	4.83	0.41	5.00	4.83
2.6	0.98	3	3.83	1.21	4	2.67	1.17	3	3.17												
2.7	0.98	3	3.83	0.75	4	3.83	0.89	4	4.00												
2.8.1	1.22	5	4.50	1.21	5	4.33	1.21	5	4.33	0.82	5.00	4.67	0.41	5.00	4.83	0.41	5.00	4.83	0.41	5.00	4.83
2.8.2	1.22	5	4.50	1.21	5	4.33	1.21	5	4.33	0.63	3.00	3.00									
Housing																					
3.1	1.17	5	3.83	0.98	4	3.17	0.98	4	3.17												
3.2	1.17	5	4.17	1.17	4	3.83	1.21	3	3.67												
3.3	0.41	4	4.17	0.55	4	3.50	1.05	3	3.50												
3.4	0.75	4	3.83	0.41	4	3.83	0.75	4	3.83												
Social Participation																					
4.1	0.41	5	4.83	0.52	5	4.67	0.52	5	4.67	0.82	5.00	4.67	0.41	5.00	4.83	0.41	5.00	4.83	0.41	5.00	4.83
4.2	0.82	5	4.33	0.75	4	4.17	0.75	4	4.17	0.63	3.00	3.00									
4.3	1.10	4	4.00	1.10	4	4.00	1.10	4	4.00	0.63	3.00	3.00									
4.4	0.98	5	4.17	0.75	4	3.83	0.63	4	4.00	0.63	3.00	3.00									
4.5	1.26	5	4.00	1.10	4	4.00	1.22	3	3.50	0.63	3.00	3.00									
4.6	0.63	4	4.00	0.55	4	3.50	0.84	3	3.50												
Respect and Social Inclusion																					
5.1	0.82	3	3.67	1.37	3	3.33	0.98	3	3.83												
5.2	1.17	5	3.83	0.98	4	3.83	1.21	5	3.67												
5.3	0.82	5	4.33	1.33	3	2.83	1.21	5	3.67												
5.4	1.51	5	3.67	1.10	3	3.00	1.33	3	3.17												
5.5	1.47	4	3.83	1.38	4	3.50	1.51	4	3.67												
Civic Participation and Employment																					
6.1	1.17	5	4.17	1.26	5	4.00	1.33	5	3.83	0.63	3.00	3.00									
6.2.1	0.41	4	3.83	1.22	4	3.50	1.17	3	3.17												
6.2.2	0.55	4	3.50	1.05	3	2.50	0.89	3	3.00												
6.3	1.17	5	3.83	1.33	4	2.83	0.98	4	3.17												
Communication and Information																					
7.1	0.89	5	4.00	0.63	4	4.00	0.84	3	3.50	0.63	3.00	3.00									
7.2	0.55	5	4.50	0.84	5	4.50	0.98	5	4.17	1.10	3.00	4.00	0.41	5.00	4.83	0.41	5.00	4.83	0.41	5.00	4.83
7.3	0.41	5	4.83	0.84	5	4.50	0.98	5	4.17	1.03	5.00	4.33	0.41	5.00	4.83	0.41	5.00	4.83	0.41	5.00	4.83
Community Support and Health Services																					
8.1	0.52	5	4.67	0.52	5	4.67	0.55	5	4.50	1.10	3.00	4.00	0.41	5.00	4.83	0.41	5.00	4.83	0.41	5.00	4.83
8.2	0.52	5	4.67	0.52	5	4.67	0.82	5	4.33	0.82	5.00	4.67	0.41	5.00	4.83	0.41	5.00	4.83	0.41	5.00	4.83
8.3	0.75	4	4.17	0.75	3	3.17	0.63	3	3.00												
8.4	0.82	5	4.33	0.75	4	4.17	0.41	4	4.17	0.63	3.00	3.00									
8.5	0.82	5	4.33	0.84	5	4.50	0.89	5	4.00	0.63	3.00	3.00									

**Table 2:** Indicators of Age-Friendliness in Taiwan’s Communities.

Category	Domain	Indicator	Description	Operational Definition		Data Source	Meaning	Implication
				Numerator	Denominator			
Core	Outdoor Space and Buildings	Proportion of public restrooms that are kept clean and accessible.	Percentage of restrooms in parks and government facilities in a township or city that have accessible toilets and entrances and are regularly cleaned.	Number of public restrooms in parks and government facilities in a township or city that have accessible toilets and are regularly cleaned.	Total number of public restrooms in parks and government agencies in a township or city.	Corroborating evidence from surveys acquired by conducting field surveys in the township or city, recording data, and using demographic statistics, health statistics, and government open data websites for verification.	The provision of adequate, convenient, and accessible restrooms would encourage older adults and those with limited physical capacity to leave their homes, facilitate physical and mental recovery, improve physical and mental health, and reduce medical and social costs.	Among the daily life functions of older adults, the process of excretion (i.e., entering and exiting the restroom independently, maintaining posture, not soiling clothes, and putting on clothes) involves complicated and challenging physical coordination. Falls are the most common type of accident among older adults. The gap between the physical response and control expectations of older adults indirectly affects their willingness to use the restroom independently. To help older adults use toilets safely and confidently, adequate, convenient, safe, and accessible restrooms must be available.
Core	Transporta tion	Availability of public transportation information.	Percentage of public transportation stations in a township and city, such as train stations, bus stations, mass rapid transit (MRT) stations, bus shelters, or bus stops that provide transportation information (e.g., paper pamphlets or Internet webpages) for people to consult.	Number of public transportation stations in a township or city (e.g., train stations, bus stations, MRT stations, bus shelters, or bus stops) that provide transportation information (portable pamphlets, Internet pages, and announcements).	Total number of public transportation stations in a township or city (train stations, bus stations, MRT stations, bus shelters, and bus stops).	Corroborating evidence from surveys acquired by conducting field surveys in the township or city, recording data, and using demographic statistics, health statistics, and government open data websites for verification.	Transportation information (timetables and routes) should be provided to older adults, and user-friendly public transportation systems should be developed to encourage older adults to move autonomously and increase their willingness to use public transportation.	Because older adults are more likely to fear or reject digital technology, they struggle to access transportation timetables in real time. When they must transfer at public transport stations, they usually need more time. Providing transportation information can help older adults use public transportation systems.
Core	Transporta tion	Satisfaction with bus drivers’ attitudes.	Percentage of older adults surveyed who considered bus drivers to be friendly in the past year.	Number of survey respondents who reported that bus drivers were always or mostly friendly over the past year (non–bus riders were excluded).	Total number of people surveyed (non–bus riders are excluded). Suggested question: “Whenever you took a bus in the past year, did you find the driver friendly toward older passengers? Were they kind? Did they wait for older adults to get off and on safely before moving driving away? Did they remind passengers to stay safe, help others, and yield seats?” 0 = “Have not taken public transportation in the past year” 1 = “Always or mostly unfriendly”; 2 = “Sometimes unfriendly”; 3 = “Mostly friendly”; 4 = “Always friendly.”	Questionnaire	Other passengers should exhibit friendly attitudes toward older adults on buses.	When older adults take buses, they often feel that drivers are unfriendly because they require more time to move on and off the bus. This may discourage older adults from taking buses.
Core	Social Participa tion	Proportion of age-friendly venues	Percentage of venues where older adults gather, such as community activity centers, community care centers, or health centers in the township or city that have adequate lighting, restrooms, and accessible facilities.	Number of venues with adequate lighting, restrooms, and accessible facilities in community activity centers, community care centers, or health centers where older adults gather in the township or city.	Total number of venues where the older adults gather, such as community activity centers, community care centers, or health centers, in the township or city.	Corroborating evidence from surveys acquired by conducting field surveys in the township or city, recording data, and using demographic statistics, health statistics, and government open data websites for verification.	Age-friendly venues (with adequate lighting and accessible facilities) should be created so that older adults have no safety concerns when participating in activities in the venues.	Places where older adults often gather for activities should be designed from the perspective of user safety and provide sufficient lighting, adequate, convenient, and safe restrooms and accessible facilities. Age-friendly venues should be created so that older adults have no safety concerns when participating in activities in the venues.
Core	Communi cation and Infor mation	Satisfaction with age-friendly counter services at government agencies.	Percentage of survey respondents who are satisfied with age-friendly counter services with designated staff or simplified services provided at government agencies (for example, township or city offices and health centers).	Number of survey respondents who are satisfied or very satisfied with age-friendly counter services or assistance provided by volunteers at government agencies (such as township and city offices and health centers).	Total number of older adults surveyed. Suggested question: “Were you satisfied with the counter services specifically for older adults and the services provided by volunteers to assist older adults when you visited government agencies (for example, township or city offices and health centers)?” 0 = “No designated counter services or volunteer assistance for older adults”; 1 = “Very dissatisfied”; 2 = “Dissatisfied”; 3 = “Satisfied”; 4 = “Very satisfied.”	Questionnaire	To provide older adults with access to special assistance at government agencies and understand how older adults feel about the consulting services offered by government agencies.	Because of declines in comprehension and perception, older adults may require special assistance to obtain information and resources.
Optional	Outdoor Space and Buildings	Proportion of public buildings that have accessible entrances and exits	Percentage of public buildings, hospitals, public markets, and activity centers in the township or city that have passages with sufficient clearance (for example, ground access, horizontal entrances, wheelchair ramps, automatic doors, and spacious passageways).	Number of public buildings, hospitals, public markets, and activity centers in the township or city that have accessibility features (such as ground access, horizontal entrances, wheelchair ramps, automatic doors, and spacious passageways)	Total number of public buildings, hospitals, public markets, and activity centers in the township or city.	Corroborating evidence from surveys acquired by conducting field surveys in the township or city, recording data, and using demographic statistics, health statistics, and government open data websites for verification.	Buildings with horizontal entrances and wheelchair ramps facilitate access for older people and those with functional disabilities.	An accessible environment removes restrictions to reduce inconvenience for individuals of all ages and enable every person to be universally respected. Public space should enable older adults and people with functional disabilities to enter and exit independently.
Optional	Transporta tion	Timely removal of road obstacles.	Percentage of road obstacles that can be removed within a reasonable amount of time after being reported by a community leader or resident.	Number of survey respondents who believe that reported road obstructions or street lighting problems (excluding those due to construction work) can be addressed within 4 weeks. Note: The 4-week criterion can be adjusted to local conditions.	Total number of people who took the survey. Suggested question: “How long does it usually take for your community to address the absence of streetlights, broken streetlights, uneven roads, or potholes (excluding problems due to road construction)?” 1 = 1–2 weeks; 2 = 2–4 weeks; 3 = 1–3 months; 4 = 3–6 months; 5 = half a year or more.	Questionnaire	Pedestrian-friendly passages or roads are flat, safe, and free of obstacles and have sufficient lighting for older adults to walk safely.	Because of the gradual deterioration of the visual and auditory functions of older adults, low-lit environments and road obstacles may cause accidents, specifically among some seniors who require mobility assistance. Pedestrian environments must enable older adults to live independently. Friendly, safe, convenient, and flat sidewalks can help older adults live independently.
Optional	Commu nity and Health Services	Proportion of care services provided at community care centers.	Percentage of community development associations, community care centers, or community leaders’ offices in the township or city that offer contact care services for those at risk of isolation.	Number of community service centers, such as community development associations, community care centers, and community leaders’ offices, in the township or city that regularly (on a fixed schedule) provide care and assistance to isolated or disabled older adults in the community.	Total number of community service organizations, such as community development associations, community care centers, and community leaders’ offices, in the township or city.	Corroborating evidence from surveys acquired by conducting field surveys in the township or city, recording data, and using demographic statistics, health statistics, and government open data websites for verification.	Community development associations, community care centers, and community leaders’ elder-care service networks help older adults leave their houses to participate in community activities.	To prevent social isolation among older adults confined to their homes, community development associations and centers and community leaders’ care service networks can help older adults establish interpersonal relationships in the community, ensure their physical and mental health, and reduce medical and social costs.
Optional	Communi cation and Infor mation	Visual friendliness satisfaction with government agencies.	Percentage of government agencies (such as township and city offices and health centers) that provide information required by the older adults clearly in large font.	Survey respondents agree or strongly agree that government agencies (such as township and city offices and health centers) provide information required by the older adults clearly in large font (those who do not visit government agencies are excluded).	Total number of older adults surveyed (excluding those who did not visit government agencies). Suggested question: “When you went to a government agency (e.g., township or city offices or health centers) to run errands in the past year, the font on the forms, documents, and indoor signage was legible.” 0 = “Haven’t been to a government agency in the past year”; 1 = “Strongly agree”; 2 = “Agree”; 3 = “Disagree”; 4 = “Strongly disagree”.	Questionnaire	Government agencies should consider older adults’ visual acuity when publishing documents so that older adults can understand them.	Older adults’ preferences and visual acuity should be accounted for to make them feel comfortable and welcome when they acquire information.
Optional	Communi cation and Infor mation	Amount of information disseminated to older adults through channels.	Percentage of health centers, community care centers, and community leaders’ offices in the township or city that disseminate health and social service information (e.g., mask purchasing and vaccination guidance) through channels such as paper pamphlets and the LINE app.	Number of health centers, community care centers, and community leaders’ offices in the township or city that disseminate health and social service information (e.g., mask purchasing and vaccination guidance) through channels such as paper pamphlets and the LINE app.	Total number of health centers, community care centers, and community leaders’ offices in the township or city.	Corroborating evidence from surveys acquired by conducting field surveys in the township or city, recording data, and using demographic statistics, health statistics, and government open data websites for verification.	Because the channels for older adults to access information vary depending on their area, multiple channels (e.g., broadcasts, telephone, and the Internet) and forms of communication (e.g., oral and written) should be used to help older adults access information.	The Internet has increased access to information and social interaction. However, older adults may fear losing sources of information and being rejected by mainstream society. Quick access to information and communication technology help older adults integrate into society.

## Data Availability

Not applicable.

## Supplementary Materials

The following supporting information can be downloaded at https://www.mdpi.com/article/10.3390/ijerph192114430/s1, Table S1: Age-Friendly Community Indicator Candidate Database.; Table S2: Quantitative changes in each stage of the development process.
