# Supplementary material for: Developing Indicators of Age-Friendliness in Taiwanese Communities through a Modified Delphi Method
Source: Int J Environ Res Public Health. 2022 Nov 4;19(21):14430. doi: 10.3390/ijerph192114430 (PMC9658640; doi:10.3390/ijerph192114430)
Supplement: Supplementary file 1 [file ijerph-19-14430-s001.zip › ijerph-1985275-supplementary.pdf]

Supplementary Information

Developing Indicators of Age-Friendliness in Taiwanese Communities Through a Modified Delphi Method

Jo-Ying Huang [1,†], Hui-Chuan Hsu [2,3,†], Yu-Ling Hsiao [4], Feng-Yin Chen [1], Shu-Ying Lo [5], Tzu-Yun Chou [5] and Megan F. Liu [1,3,*]

[1] School of Gerontology and Long-Term Care, College of Nursing, Taipei Medical University, Taipei 11031, Taiwan
[2] School of Public Health, College of Public Health, Taipei Medical University, Taipei 11031, Taiwan
[3] Research Center of Health Equity, College of Public Health, Taipei Medical University, Taipei 11031, Taiwan
[4] Department of Nursing, Center of Geriatric Care Resource, College of Medicine, Fu Jen Catholic University, Taipei 242062, Taiwan
[5] Division of Community Health Division, Health Promotion Administration, Ministry of Health and Welfare, Taipei 103205, Taiwan
* Correspondence: fangliu@tmu.edu.tw; Tel.: +886-2-27361661-6331 (ext. 6341)
† These authors contributed equally to this work.

Supplementary Table S1: Indicator candidates

Domain	Guidelines for Age-Friendly Communities in Taiwan	Draft of Indicators		
		Description	Data source*	Operational definition
Outdoor Space and Buildings	1.1 Green space, plenty of outdoor seating, rest areas, and awnings must be provided, and the safety of the space must be ensured.	1.1 The green space in the community (communities must have a certain amount of green space) should have outdoor seating, rest areas, and awnings, and parks should be safe.	Corroborating evidence from survey	The percentage of parks in the area with benches, seating areas, awnings, and safety measures.
	1.2 Safe and accessible sidewalks (flat, spacious, well-lit, crack-less, and antislip) must be maintained, and pedestrian priority lanes and bicycle lanes should be separated.	1.2.1 Proportion of sidewalks and arcades that are safe and accessible (even, no cracks, antislip).	Corroborating evidence from survey	The percentage of sidewalk sections and arcades that are safe and accessible (even, no cracks, antislip).
		1.2.2 Proportion of pedestrian priority lanes and separate lanes for pedestrians and vehicles.	Corroborating evidence from survey	Pedestrians have priority lanes and separate lanes from those for vehicles near schools, hospitals, and senior facilities in the area.
	1.3 Pedestrian crosswalks must have safety features that are accessible to older adults and people with disabilities (e.g., antislip designs, appropriate traffic signal cycle length, connections between crosswalks and pedestrian crossing islands, high visibility at intersections, and legible electronic signage).	1.3 All traffic lights have sufficiently long cycle lengths and are clearly marked.	Questionnaire	No traffic lights in the vicinity have insufficient cycle lengths or are unclearly marked (yes/no).
	1.4 Public buildings must have adequate and wheelchair-friendly passageways outside the facilities for ingress and egress (e.g., ground passages, horizontal entrances, accessible elevators, wheelchair ramps, automatic doors, spacious passages).	1.4 Public buildings have adequate and wheelchair-friendly passageways outside the buildings for ingress and egress (e.g., ground passages, horizontal entrances, accessible elevators, wheelchair ramps, automatic doors, spacious passages).	Corroborating evidence from survey	Proportion of government agencies, hospitals, markets, and activity centers in the district that have a sufficient number of wheelchair-friendly passageways outside the buildings for ingress and egress (e.g., ground passages, horizontal entrances, accessible elevators, wheelchair ramps, automatic doors, spacious passages).
	1.5 Indoor and outdoor public restrooms have an adequate number of accessible toilets that	1.5 Public restrooms in parks and public buildings have toilets with accessible entrances that are kept clean.	Corroborating evidence from survey	Proportion of public restrooms in parks and public buildings in the district that have accessible toilets and

Domain	Guidelines for Age-Friendly Communities in Taiwan	Draft of Indicators		
		Description	Data source*	Operational definition
	are kept clean and easily accessible for older adults.			accessible entrances and that are cleaned daily.
	2.1 Local businesses should sponsor community shuttles to shopping and other business centers.	2.1 Shuttles or minibuses to shopping centers or hospitals in remote communities are provided.	Corroborating evidence from survey	Proportion of remote townships and cities that have shuttles or minibuses to shopping centers or hospitals.
	2.2 Public transportation is well connected throughout the entire district, and the routes and vehicles are clearly marked.	2.2 Public transportation is provided throughout the entire district.	Corroborating evidence from survey	Percentage of cities and townships reachable through public transportation.
	2.3 Transportation information, including routes, timetables, and facilities are provided for people with special needs.	2.3 Public transportation information for buses, trains, and ferries is easily obtainable.	Corroborating evidence from survey	Proportion of bus stations, train stations, and ports in the city that provide transportation information (signage, pamphlets, or the Internet).
	2.4 Bus shelters are conveniently located, accessible, safe, clean, well-lit, and have clear signage, adequate seating, and covered space.	2.4 Bus shelters have covered space for resting and have clear signage.	Corroborating evidence from survey	Percentage of bus shelters in the district that have covered space for resting and have clear signage.
Transportation	2.5 Roads are always maintained and have proper lighting, accessible passages, traffic safety equipment (e.g., speed bumps), and clear signs to prevent confusion.	2.5 Roads are well-lit and are free of obstacles.	Questionnaire	Proportion of road obstacles removed in a reasonable amount of time after being reported by community leaders or residents.
	2.6 Regular training and refresher courses are provided to senior drivers.	2.6 Transportation service companies offer training courses (as required by the county and city government).	Corroborating evidence from survey	Percentage of transportation service companies that offer periodic training courses to senior drivers.
	2.7 Road safety awareness in local senior communities is raised through campaigns and education.	2.7 Public health centers or communities hold seminars and campaigns about road safety.	Corroborating evidence from survey	Local public health centers and communities conduct road safety seminars and activities every year (yes/no).
	2.8 The attitudes of mass transit drivers toward older adults are improved through campaigns and training.	2.8.1 The public rates the friendliness of mass transit drivers toward older adults.	Questionnaire	Questionnaire: Number of people who think drivers were friendly when taking mass transit over the past year / number of older adults surveyed.
		2.8.1 The public rates the friendliness of mass transit station crew members toward older adults.	Questionnaire	Number of older adults who think station crew members have been friendly to them in the past year

Domain	Guidelines for Age-Friendly Communities in Taiwan	Draft of Indicators		
		Description	Data source*	Operational definition
				divided by the number of older adults surveyed.
Housing	3.1 Affordable and adequate home care services are provided to older adults with physical or mental disabilities.	3.1 Proportion of senior social housing to senior population size and the average time required to get off the waiting list for nursing homes.	Corroborating evidence from survey	(1) Proportion of social housing to the number of older adults who have no self-occupied houses (data provided by the Social Affairs Bureau); (2) Wait time to get into nursing homes (provided by nursing institutions).
	3.2 Service networks are provided for home repair and support.	3.2 Presence of a department in the district that provides home repairs for older adults.	Corroborating evidence from survey	Proportion of house renovation and repair companies that can provide services to older adults in the district who lack mobility.
	3.3 Options for older adults to age at home are available, and information on services such as housing renovation and maintenance programs is provided.	3.3 Presence of a designated department in the district that provides services and information on home renovation and maintenance plans.	Corroborating evidence from survey	Channels for older adults to acquire information on home repair and maintenance subsidies (city government webpage, LINE app, community leaders, or long-term care case managers).
	3.4 Crime (including fraud and abuse) prevention strategies and programs are provided to older adults.	3.4 Campaigns to promote fraud prevention and abuse reporting.	Corroborating evidence from survey	Public health centers, community centers, or village chiefs have conducted fraud prevention and abuse reporting campaigns in the past year (yes/no).
Social Participation	4.1 Venues where community activities are held should be brightly lit, have adequate restrooms, and be easily accessible via public transportation.	4.1 Percentage of community activity centers, community care centers, health centers, and meeting venues that have achieved these goals.	Corroborating evidence from survey	Percentage of community activity centers, community care sites, health centers, and meeting venues that are well-lit and have adequately equipped restrooms.
	4.2 Events are held during times when older adults can participate.	4.2 The schedule for community activities is suitable for older adults.	Questionnaire	Percentage of older adults who believe that community activities are scheduled at an appropriate time.

Domain	Guidelines for Age-Friendly Communities in Taiwan	Draft of Indicators		
		Description	Data source*	Operational definition
	4.3 The format and content of community activities enable older adults to participate alone or have others accompany them.	4.3 Community activities can be attended by older adults alone or with others.	Questionnaire	Percentage of older adults who believe that community activities can be attended alone or with others.
	4.4 Care services and mutual assistance networks are provided to vulnerable older adults, such as those who live alone.	4.4 Community leaders and community care centers provide information regarding National Health Insurance, social welfare, subsidies, hospital and clinic palliative care, hospice services, advance directives, and legal consultation regarding the Patient Right to Autonomy Act for people living alone and disadvantaged individuals. Family care, counseling, and assistance mechanisms are provided to families of the deceased.	Corroborating evidence from survey	Percentage of community leaders and community centers that have mechanisms to provide care and individualized information for disadvantaged individuals.
	4.5 Channels are provided to older adults to express their opinions and encourage them to participate in activity decision-making and planning.	4.5 Channels such as neighborhood resident assemblies and community management committee meetings are available for older adults to express their opinions	Corroborating evidence from survey	Percentage of villages that have resident assemblies, community management committee meetings, or other channels for residents to express opinions.
	4.6 Courses and networking opportunities are provided for family members and caregivers of older adults.	4.6 Community care centers and family caregiver service centers provide caregiving courses and networking opportunities.	Corroborating evidence from survey	Proportion of townships and cities that have family caregiver service centers.
Respect and Social Inclusion	5.1 Older adults receive positive representation in media campaigns.	5.1 Counties and cities organize activities for older adults and release photos or reports projecting a positive image of older adults.	Corroborating evidence from survey	Proportion of counties and cities that organize activities for older adults and release photos or reports projecting a positive image of older adults (for example, senior volunteer awards, grandpa and grandma vitality health shows).
	5.2 Service-oriented activities in the community attract participants of all generations and account for the special needs and preferences of older adults.	5.2 Community activities are designed to encourage intergenerational participation and include at least one activity specifically for older adults.	Corroborating evidence from survey	Percentage of communities (villages or community centers) that hold intergenerational activities.

Domain	Guidelines for Age-Friendly Communities in Taiwan	Draft of Indicators		
		Description	Data source*	Operational definition
	5.3 Schools offer courses that recognize older adults and arrange for older adults to become part of curriculum activities.	5.3 The curriculum of primary and secondary schools includes teaching activities on topics related to older adults (at least once a year).	Corroborating evidence from survey	Proportion of primary and secondary school curricula or activities in the county and city that include older adult–related topics at least once a year (for example, outdoor learning activities or visits organized by long-term care institutions; grandparent festivals).
	5.4 Dementia-friendly hospitals.	5.4 Patients with dementia who seek medical treatment for diseases other than dementia receive the same or more assistance than do other patients.	Questionnaire	Percentage of people who believe that patients with dementia seeking medical treatment for diseases other than dementia at health centers or medical institutions receive the same quality of care as do other patients ("strongly disagree" to "strongly agree").
	5.5 Government agencies create a dementia-friendly environment.	5.5 Government employees are friendly to patients with dementia.	Questionnaire	Government agencies (such as health centers and district offices) are friendly to patients with dementia ("strongly disagree" to "strongly agree").
Civic Participation and Employment	6.1 Various volunteer service opportunities are provided.	6.1 The proportion of volunteer service departments that provide a range of volunteering opportunities to senior volunteers.	Corroborating evidence from survey	Proportion of volunteer services departments in the district that provide senior volunteers with a range of volunteering opportunities.
	6.2 Various paid job opportunities and training are provided to older adults.	6.2.1 Proportion of businesses and institutions offering flexible work or part-time jobs to older adults.	Corroborating evidence from survey	Number of stores employing people aged older than 55 years for flexible or part-time work.
		6.2.2 Number of businesses providing employment training guidance.	Corroborating evidence from survey	The proportion of older adults who receive employment training.
	6.3 Postretirement training courses are provided to older employees.	6.3 Senior citizen learning camps, enterprises, and community colleges provide postretirement training courses to older employees.	Corroborating evidence from survey	Number of senior employee postretirement training courses offered

Domain	Guidelines for Age-Friendly Communities in Taiwan	Draft of Indicators		
		Description	Data source*	Operational definition
				by senior colleges, enterprises, and community colleges in the district.
Communication and Information	7.1 Services that support oral communication with older adults are available.	7.1 Amount of information disseminated orally by community leaders.	Questionnaire	Percentage of people agreeing that community leaders verbally explain key information or that community leaders would offer verbal explanations in their offices (“strongly disagree” to “strongly agree”).
	7.2 One-on-one communication with older adults at risk of social isolation (such as those who live alone) is available.	7.2 Community development associations, centers, or neighborhood chiefs provide a network of care services for those at risk of isolation.	Corroborating evidence from survey	Proportion of community development associations, centers, or community leaders who provide contact care services to people at risk of isolation.
	7.3 Information and documents required by older adults have enlarged text and simple and clear headings and use bold, black font to display crucial information.	7.3 Government agencies (such as health centers) provide older adult–specific information clearly and in large font.	Questionnaire	Percentage of people who agree that government agencies (such as district offices and health centers) provide age-friendly versions of information clearly and with large font (“strongly disagree” to “strongly agree”).
Community Support and Health Services	8.1 Older adults have access to health and social services.	8.1 Channels to provide information to older adults are available (e.g., pamphlets, flyers, community leaders’ LINE accounts, and broadcasts).	Corroborating evidence from survey	Health centers, community leaders’ offices, and community service centers provide paper flyers, LINE quick-response codes, and other channels for health and social service information (e.g., mask purchases and flu shots).
	8.2 The administrative procedures of public services are simplified for older adults.	8.2 Hospitals and government agencies have age-friendly counters with assigned staff and simplified processes.	Questionnaire	Level of satisfaction with services provided by staff at age-friendly counters or volunteers at government agencies (such as district offices and health centers; “very dissatisfied” to “very satisfied”).

Domain	Guidelines for Age-Friendly Communities in Taiwan	Draft of Indicators		
		Description	Data source*	Operational definition
	8.3 Community emergency plans account for the capacities and mobility of older adults.	8.3 Disaster prevention and contingency measures include special consideration for older adults (yes/no).	Corroborating evidence from survey	County and city governments or townships and city offices consider older adults in disaster prevention and response measures (such as the evacuation of villages, transportation assistance, and resettlement).
	8.4 Individualized life and health service resources are available.	8.4 Amount of individualized service resource information provided by community leaders and community care centers to residents (regarding National Health Insurance, social welfare, subsidies, hospital and clinic palliative care and hospice services, advanced directives, the Patient Right to Autonomy Act, and legal counseling to patients living alone and disadvantaged individuals; counseling and assistance mechanisms for families of the deceased.)	Corroborating evidence from survey	Percentage of village chiefs and community centers that provide care and individualized information to disadvantaged individuals.
	8.5 Community health service resource information is regularly updated.	8.5 Health centers regularly update health service information on bulletin boards.	Corroborating evidence from survey	Health centers regularly update health service information on bulletin boards (monthly).

*Corroborating evidence from surveys refers to public data collected during field visits by the government. Questionnaires were administered by health centers or the communities.

Supplementary Table S2: Quantitative changes in each stage of the development process.

Domain	Candidates	First Round		Second Round		Third Round		External Review		Final
	Quantity	Revised<br>Added<br>Deleted	Quantity	Revised<br>Added<br>Deleted	Quantity	Revised<br>Added<br>Deleted	Quantity	Revised<br>Added<br>Deleted	Quantity	Quantity
		3		2		2		1		
Outdoor Space and Buildings	6	0	3	0	2	0	2	0	2	2
		3		1		0		0		
		6		3		3		2		
Transportation	9	0	6	0	3	0	3	0	3	3
		3		3		0		0		
		0		0		0		0		
Housing	4	0	0	0	0	0	0	0	0	0
		4		0		0		0		
		5		1		1		1		
Social Participation	6	0	5	0	1	0	1	0	1	1
		1		4		0		0		
		0		0		0		0		
Respect and Social Inclusion	5	0	0	0	0	0	0	0	0	0
		5		0		0		0		
		1		0		0		0		
Civic Participation and Employment	4	0	1	0	0	0	0	0	0	0
		3		1		0		0		
		3		2		2		1		
Communication and Information	3	0	3	0	2	0	2	1	1	2
		0		1		0		1		
		4		2		2		1		
Community Support and Health Services	5	0	4	0	2	0	2	1	1	2
		1		2		0		1		
**Total**	42		22		10		10		10	10
